# A novel fungal GH30 xylanase with xylobiohydrolase auxiliary activity

**DOI:** 10.1186/s13068-019-1455-2

**Published:** 2019-05-11

**Authors:** Constantinos Katsimpouras, Grigorios Dedes, Nikolaos S. Thomaidis, Evangelos Topakas

**Affiliations:** 1 Industrial Biotechnology & Biocatalysis Group, School of Chemical Engineering, National Technical University of Athens, 9 Iroon Polytechniou Str., Zografou Campus, 15780 Athens, Greece; 2 Laboratory of Analytical Chemistry, Department of Chemistry, National and Kapodistrian University of Athens, Panepistimioupolis Zografou, 15771 Athens, Greece; 3 Biochemical and Chemical Process Engineering, Division of Sustainable Process Engineering, Department of Civil, Environmental and Natural Resources Engineering, Luleå University of Technology, 97187 Luleå, Sweden

**Keywords:** GH30 xylanase, Xylooligosaccharides, *Thermothelomyces thermophila*, Glucuronoxylan, Xylobiohydrolase

## Abstract

**Background:**

The main representatives of hemicellulose are xylans, usually decorated β-1,4-linked d-xylose polymers, which are hydrolyzed by xylanases. The efficient utilization and complete hydrolysis of xylans necessitate the understanding of the mode of action of xylan degrading enzymes. The glycoside hydrolase family 30 (GH30) xylanases comprise a less studied group of such enzymes, and differences regarding the substrate recognition have been reported between fungal and bacterial GH30 xylanases. Besides their role in the utilization of lignocellulosic biomass for bioenergy, such enzymes could be used for the tailored production of prebiotic xylooligosaccharides (XOS) due to their substrate specificity.

**Results:**

The expression of a putative GH30_7 xylanase from the fungus *Thermothelomyces thermophila* (synonyms *Myceliophthora thermophila*, *Sporotrichum thermophile*) in *Pichia pastoris* resulted in the production and isolation of a novel xylanase with unique catalytic properties. The novel enzyme designated *Tt*Xyn30A, exhibited an endo- mode of action similar to that of bacterial GH30 xylanases that require 4-*O*-methyl-d-glucuronic acid (MeGlcA) decorations, in contrast to most characterized fungal ones. However, *Tt*Xyn30A also exhibited an exo-acting catalytic behavior by releasing the disaccharide xylobiose from the non-reducing end of XOS. The hydrolysis products from beechwood glucuronoxylan were MeGlcA substituted XOS, and xylobiose. The major uronic XOS (UXOS) were the aldotriuronic and aldotetrauronic acid after longer incubation indicating the ability of *Tt*Xyn30A to cleave linear parts of xylan and UXOS as well.

**Conclusions:**

Hereby, we reported the heterologous production and biochemical characterization of a novel fungal GH30 xylanase exhibiting endo- and exo-xylanase activity. To date, considering its novel catalytic properties, *Tt*Xyn30A shows differences with most characterized fungal and bacterial GH30 xylanases. The discovered xylobiohydrolase mode of action offers new insights into fungal enzymatic systems that are employed for the utilization of lignocellulosic biomass. The recombinant xylanase could be used for the production of X2 and UXOS from glucuronoxylan, which in turn would be utilized as prebiotics carrying manifold health benefits.

**Electronic supplementary material:**

The online version of this article (10.1186/s13068-019-1455-2) contains supplementary material, which is available to authorized users.

## Background

Hemicelluloses are heterogeneous polysaccharides consisting of a β-1,4-linked backbone of pentoses, hexoses, and sugar acids, which are usually decorated with side branches [1]. The primary hemicelluloses are xylans, mannans, arabinans, and galactans. The exact structure and abundance of hemicelluloses diversify widely among different plant species and cell types with xyloglucans occurring mostly in the primary walls of dicots and conifers, while arabinoxylans dominate in commelinid monocots [2]. Softwoods contain galactoglucomannans, arabinoglucuronoxylans, arabinogalactans, xyloglucans, and other glucans. The leading representative of hardwood hemicellulose is xylan and more specifically *O*-acetyl-4-*O*-methylglucuronoxylan, which accounts for 80% to 90% of hardwood hemicelluloses. Hemicelluloses are also present in most agricultural wastes such as wheat straw, rice straw, corn stover, and sugar cane bagasse among others. Agricultural and forest residues have the potential to serve as a sustainable source of sugars that can be subsequently used as feedstock for the production of biofuels or high added-value bio-based materials.

The enzymatic breakdown of xylans, the main representatives of hemicelluloses, requires the action of a group of enzymes with various specific activities such as xylanases and β-xylosidases. The enzymes that are responsible for the degradation of xylan main chain are endoxylanases (E.C. 3.2.1.8) and they have been grouped in glycoside hydrolases families 5, 8, 10, 11, 16, 30, 43, 51, 98, and 141 of the CAZy database (http://www.cazy.org; Lombard et al. [3]) based on amino acid similarities and structural characteristics. Xylanases belonging to GH10 and GH11 families have been widely investigated in literature with GH10 xylanases exhibiting less substrate specificity than GH11 xylanases. Therefore, the former enzymes are able to catalyze the hydrolysis of a wide spectrum of xylans, while the latter are known to preferentially cleave unsubstituted regions of arabinoxylan [4].

A less studied group of endoxylanases belongs to GH30 family. Family GH30 of the glycosyl hydrolases exhibits a wide diversity. Enzymes belonging to this family include β-glucosidases (3.2.1.21), β-glucuronosidases (EC 3.2.1.31), β-xylosidases (3.2.1.37), β-fucosidases (EC 3.2.1.38), glucosylceramidases (EC 3.2.1.45), β-1,6-glucanases (EC 3.2.1.75), glucuronoarabinoxylan endo-β-1,4-xylanase (EC 3.2.1.136), and endo-β-1,6-galactanase (EC 3.2.1.164). Furthermore, GH30 family comprise 8 subfamilies of which the subfamily 8 includes enzymes that have been shown to be appendage-dependent since they act against xylan decorated with 4-*O*-methyl-d-glucuronic acid (MeGlcA) side substituents [5, 6]. Bacterial xylanases such as XynA from *Erwinia chrysanthemi* and XynC from *Bacillus subtilis* belong to GH30_8 family and they appeared to be functional against glucuronoxylan, while these enzymes do not act, or act orders of magnitude slower, against oligosaccharides or polysaccharides that are not decorated with MeGlcA [7–9]. Similar GH30_8 xylanases that have been characterized are Xyn5B from *Bacillus* sp. strain BP-7, Xyn30D from *Paenibacillus barcinonensis*, *Bs*Xyn30 from *Bacillus subtilis* LC9, and *St*Xyn30A from *Streptomyces turgidiscabies* [10–13]. However, the *Cp*Xyn30A from *Clostridium papyrosolvens* has been reported to hydrolyze glucuronoxylan, neutral xylooligosaccharides and wheat arabinoxylan, probably due to a loss of amino acid sequence conservation in a region involved in glucuronic acid (GlcA) recognition [14]. Moreover, a GH30_8 xylanase from *Clostridium acetobutylicum* was reported as a GlcA-independent endo-xylanase that shows preference against arabinofuranose-substituted xylan chains [15]. GH30 xylanases of fungal origin have also been shown to differ compared to those from bacteria as they exhibit a broader substrate specificity. The xylanases XynD from *Bispora* sp. and XYN IV from *Trichoderma reesei* have been reported to be active also on arabinoxylan, thus exhibiting different catalytic properties from bacterial GH30 xylanases [16, 17]. Nevertheless, Biely et al. [5] described the expression of a novel GH30 xylanase from *T. reesei* designated XYN VI that exhibited catalytic properties almost identical to bacterial GH30 xylanases [5].

The specificity of GH30 endoxylanases renders them a key tool for the generation of uronic xylooligosaccharides (UXOS) that could function as prebiotic compounds and preserve a balanced intestinal microflora. Endoxylanases, in general, comprise significant tools for the production of tailored, prebiotic oligosaccharides [18]. Production of UXOS from biomass has been reported from sweet sorghum and sweetgum wood, using GH11 and GH30 xylanases from *Bacillus subtilis* [19, 20]. Moreover, Valls et al. [21] investigated the antioxidant activity of XOS produced from beechwood glucuronoxylan using a GH30 xylanase from *P. barcinonensis* and found out that it was significantly higher than that from XOS produced by a GH10 xylanase.

Enzymes from thermophilic microorganisms are of great biotechnological and industrial interest as they exhibit improved robustness and are able to endure more severe process conditions [22]. *Thermothelomyces thermophila* (synonyms *Myceliophthora thermophila*, *Sporotrichum thermophile*) is a thermophilic fungus that possesses an impressive arsenal of hemicellulolytic enzymes, which are necessary to overcome the recalcitrance of plant cell wall. The sequencing and annotation of *T. thermophila* genome revealed a great number of putative cell wall-degrading enzymes [23]. Ten xylanases, with four of them belonging to GH10 family and the rest to GH11 family, have been purified and characterized from multienzyme preparations of *T. thermophila* modified strains [24, 25]. Two genes encode putative GH30 xylanolytic enzymes, yet the characterization of a GH30 xylanase from *T. thermophila* has not been conducted [23].

In the present study, we reported the cloning of the *T. thermophila ex30a* gene and its heterologous expression using the *P. pastoris* expression system. The biochemical characterization and determination of the recombinant enzyme’s catalytic properties were conducted as well. The findings revealed that the gene codes for a *T. thermophila* GH30 xylanase, dubbed *Tt*Xyn30A, which functions not only as an appendage-dependent GH30 endo-xylanase but also shows exo-xylanase activity from the non-reducing end liberating xylobiose (X2) from linear and substituted xylan chains. During the publication process of the present study, a novel GH30_7 xylanase from the cellulolytic fungus *Talaromyces cellulolyticus* has been reported to possess both endo-glucuronoxylan and exo-xylobiohydrolase activities, confirming the discovered unique mode of action [26].

## Results

### Sequence analysis of *Tt*Xyn30A

The putative endo-xylanase gene encodes a protein of 477 amino acids including a secretion signal peptide of 19 amino acids (MYSLLIALLCAGTAVDAQA) as it was predicted by SignalP 4.0 server. A sequence analysis using the InterProScan server showed that the enzyme involves a GH30 catalytic domain, while it is not modular. The mature protein comprises 458 amino acids and the theoretical MW and p*I* were calculated using the ExPASY ProtParam tool and were found to be 48,976.2 Da and 5.5, respectively. The putative xylanase was cloned and expressed using the gene *ex30a*, which was synthesized as codon optimized for expression in *P. pastoris* omitting the signal peptide and its 3 introns.

The sequence homology values between the mature protein *Tt*Xyn30A and other GH30 xylanases, either of bacterial or fungal origin, were calculated and are presented in Table 1. The putative xylanase exhibited amino acid sequence identity from 22 to 24% and similarity from 40 to 45% in comparison with bacterial GH30 xylanases. Higher homology was exhibited compared with fungal GH30 xylanases. *Tt*Xyn30A showed amino acid sequence identities of 39.7%, 38.5%, 44.2%, and 43.8% with four xylanases from microorganisms *Bispora* sp. MEY-1, *T. reesei*, *T. cellulolyticus*, and *Penicillium purpurogenum*, respectively (59.2%, 53.9%, 61.8%, and 62.5% similarity, respectively). The XYN VI from *T. reesei* [5] was the closest characterized sequence homolog with 44.3% sequence identity (61.4% sequence similarity).

**Table 1:** Identity and similarity values among the amino acid sequences of the mature xylanase *Tt*Xyn30A and other mature GH30 xylanases from different microorganisms (carbohydrate binding domains or dockerins are not included)

Microorganism (GH30 xylanase)	Identity	Similarity
Bacterial		
*Aeromonas caviae* (XynD)	23.4% (85/363)	44.9% (163/363)
*Bacillus* sp. BP7 (Xyn5B)	22.1% (76/344)	40.4% (139/344)
*Bacillus subtilis* 168 (XynC)	22.4% (77/344)	38.7% (133/344)
*Clostridium papyrosolvens* (CpXyn30A)	22.4% (76/339)	44.8% (152/339)
*Clostridium thermocellum* (CtXyn30A)	22.9% (75/327)	41.9% (137/327)
*Erwinia chrysanthemi* (XynA)	24.3% (111/456)	39.7% (181/456)
*Paenibacillus barcinonensis* (Xyn30D)	24.2% (90/372)	42.2% (157/372)
*Streptomyces turgidiscabies* C56	23.2% (73/315)	42.9% (135/315)
Fungal		
*Bispora* sp. MEY-1 (XylD)	39.7% (180/453)	59.2% (268/453)
*Penicillium purpurogenum* (XynC)	43.8% (201/459)	62.5% (287/459)
*Talaromyces cellulolyticus* (Xyn30B)	44.2% (201/455)	61.8% (281/455)
*Trichoderma reesei* (XYN IV)	38.5% (153/397)	53.9% (214/397)
*Trichoderma reesei* (XYN VI)	44.3% (200/451)	61.4% (277/451)

### Heterologous expression and physicochemical properties of *Tt*Xyn30A

**Fig. 1:**
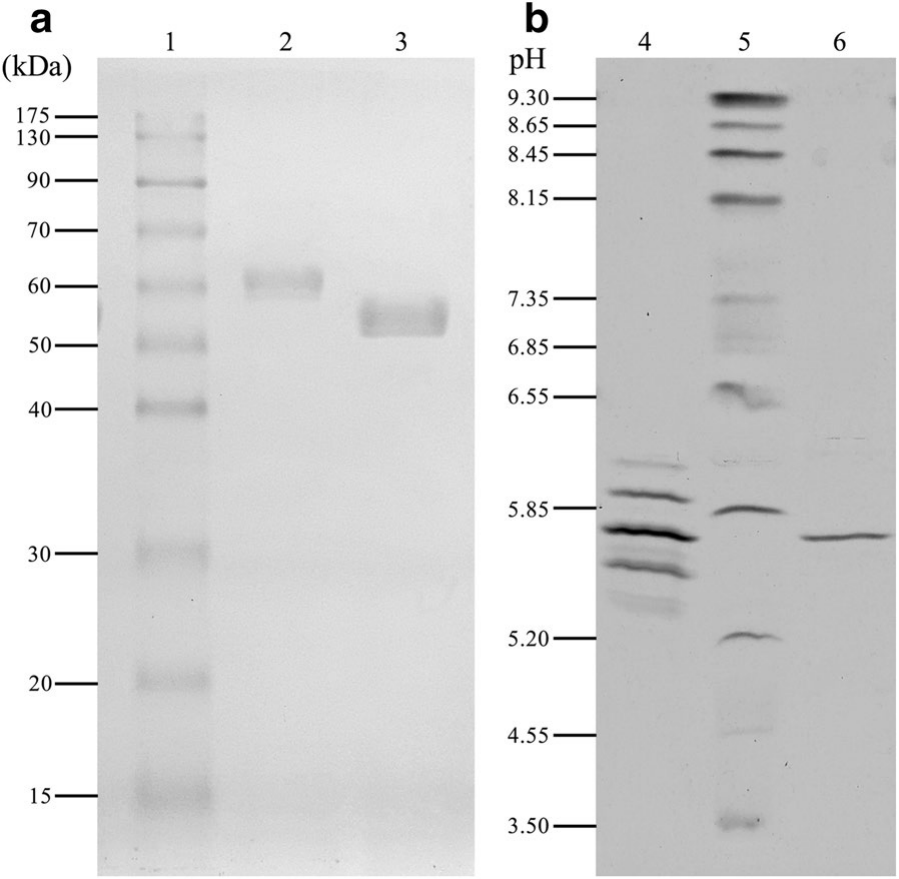
SDS-PAGE (**a**) and IEF (**b**) of *Tt*Xyn30A. **a** LMW standard protein markers (1), purified *Tt*Xyn30A (2), and *Tt*Xyn30A after enzymatic treatment with EndoH (3). **b** Purified *Tt*Xyn30A (4), standard protein markers with p*I* range 3.0–10.0 (5), and *Tt*Xyn30A after enzymatic treatment with EndoH (6)

The purified xylanase *Tt*Xyn30A exhibited an estimated MW of 61 kDa as indicated by SDS-PAGE analysis (Fig. 1a). Furthermore, the p*I* of *Tt*Xyn30A was experimentally determined by IEF-PAGE and was found to be in the range of 5.3–6.2 (Fig. 1b). The MW of the purified *Tt*Xyn30A was higher than the calculated value of 51.7 kDa, including the myc epitope and the polyhistidine tag that contribute to the increase of the MW by about 2.5 kDa. One N-glycosylation site was predicted using the NetNGlyc 1.0 server at residue N176 and might be responsible for the observed difference in MW of *Tt*Xyn30A. Additionally, four potential O-glycosylation sites were identified using the NetOGlyc 4.0 server (3 Ser and 1 Thr). The MW of the recombinant xylanase decreased by about 9 kDa after treatment with Endoglycosidase H (EndoH), which is an indication of N-glycosylation (Fig. 1a).

### Biochemical characterization of purified *Tt*Xyn30A

*Tt*Xyn30A optimum conditions were investigated using beechwood glucuronoxylan as substrate. The optimum activity was observed at pH 4.0, while the enzyme maintained more than 88% of its initial activity at pH range from 3.5 to 4.5 (Additional file 1: Figure S1a). Moreover, *Tt*Xyn30A maintained about 77% of its initial activity at pH 5.0, while a sharp loss in enzyme’s activity was noted at pH 3.0 and at pH values greater than 5.0 (10% of its initial activity at pH 6.0). The xylanase was stable at pH range from 3.0 to 9.0 after a 24-h incubation, maintaining more than 97% of its initial activity (Additional file 1: Figure S2).

Optimal temperature was achieved at 50 °C and pH 4.0, while the enzyme lost rapidly its activity at temperatures over 55 °C (Additional file 1: Figure S1b). In addition, *Tt*Xyn30A was adequately active even at 40 °C and 45 °C, where it retained more than 70% and 88% of its initial activity, respectively. Beechwood xylan hydrolysis rate at 50 °C and pH 4.0 is shown in Additional file 1: Figure S3. A linear increase of the hydrolysis rate was observed for the first 4 h, indicating the stability of the recombinant xylanase at the optimum temperature. When it comes to the hydrolysis rate of wheat arabinoxylan, it was orders of magnitude lower than that achieved during glucuronoxylan hydrolysis.

The specific activity of recombinant *Tt*Xyn30A was investigated using a variety of substrates at 50 °C and pH 4.0. The highest specific activity was observed with beechwood glucuronoxylan and was found to be 6 U mg^−1^. Against wheat arabinoxylan the specific activity was much lower (0.07 U mg^−1^), indicating a preference for substrates substituted with MeGlcA. The enzyme showed no activity against carob galactomannan, konjac glucomannan, and barley β-glucan. The recombinant *Tt*Xyn30A exhibited a catalytic efficiency *k*_cat_/*K*_m_ of 254.8 mL mg^−1^ min^−1^ (*K*_m_= 1.7 ± 0.1 mg mL^−1^ and *V*_max_= 7.1 ± 0.2 U mg^−1^).

The presence of Co^2+^, Cu^2+^, and Mn^2+^ enhanced the activity as high as 44% at concentration of 10 mM. Furthermore, a partial inhibition, from 5 to 15%, of the enzyme was observed in the presence of certain metal ions or chemical compounds at the concentration of 10 mM (from higher inhibition to lower): EDTA > Mg^2+^ > Urea, Zn^2+^ > Fe^3+^. The presence of the rest of the metal ions had little on negligible effect (Additional file 1: Table S1).

### Hydrolysis product analysis

**Fig. 2:**
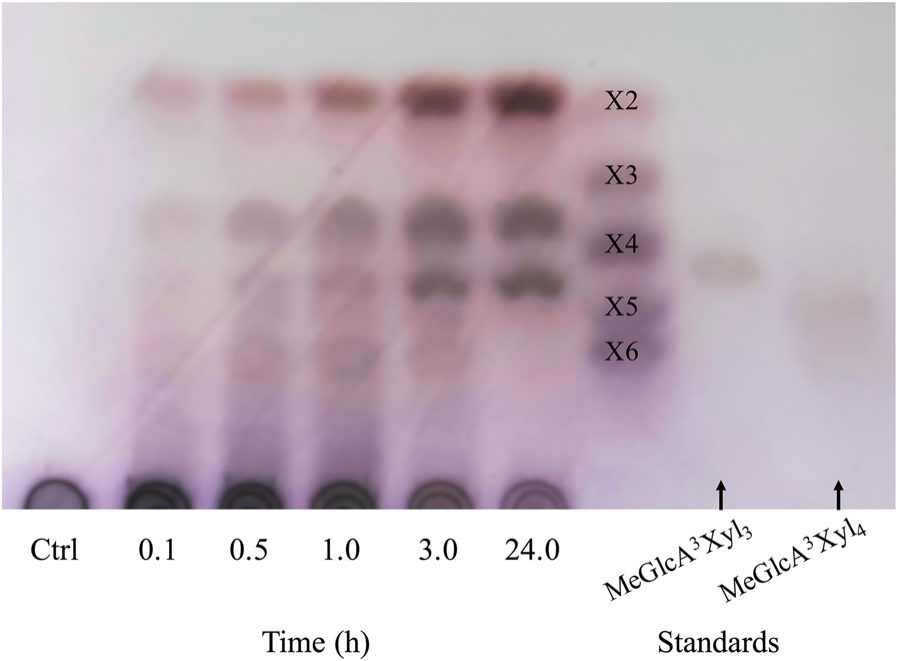
TLC analysis of hydrolysis products from beechwood xylan by recombinant *Tt*Xyn30A. The reaction was carried out in 0.05 mM citrate–phosphate buffer pH 4.0 at 50 °C. The substrate and enzyme loadings were 5 mg mL^−1^ and 0.09 U mL^−1^, respectively. Xylooligosaccharides (DP 2–6) and aldouronic acids (aldotetrauronic and aldopentauronic acid) were used as standards

**Fig. 3:**
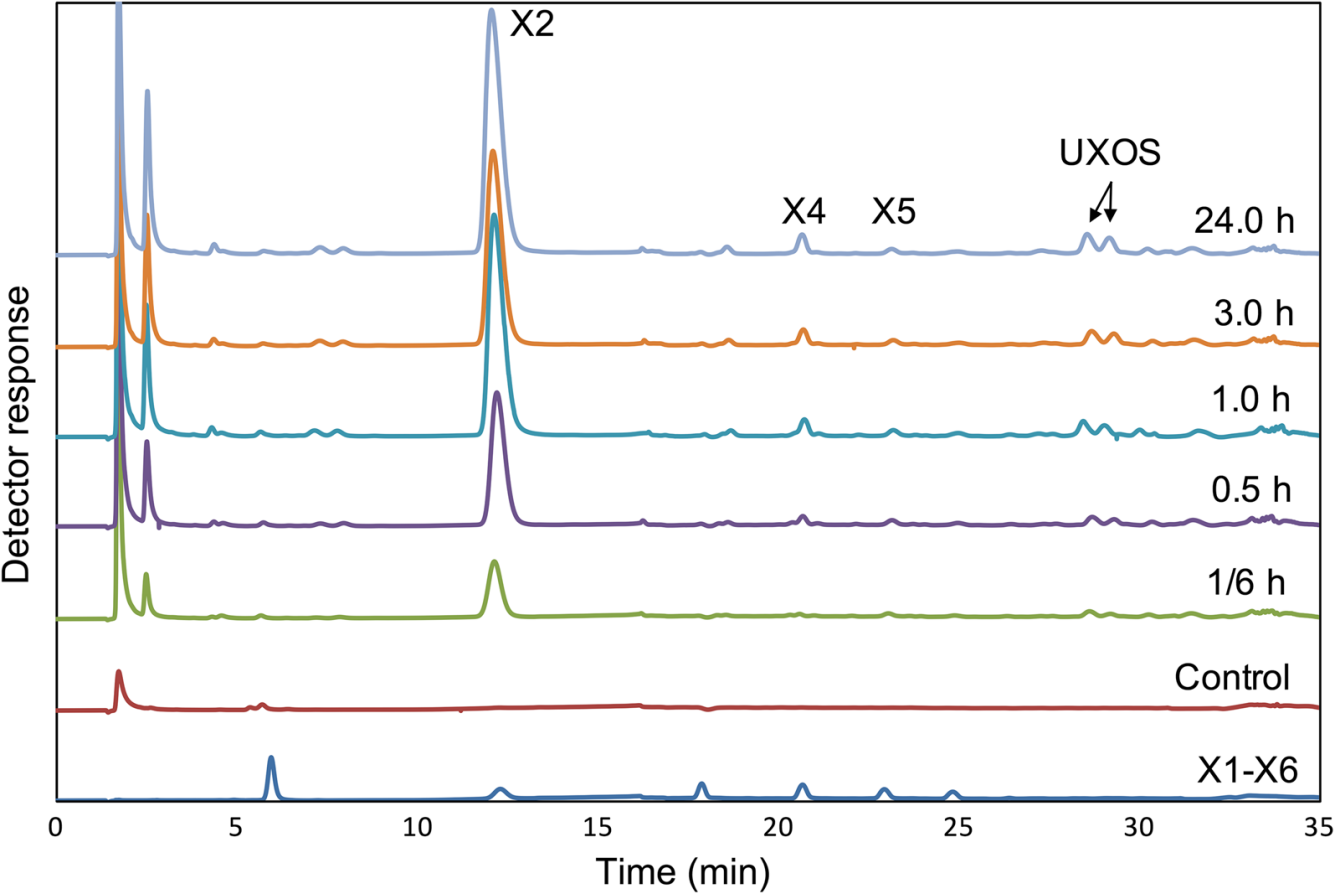
Time course of the hydrolysis products of beechwood glucuronoxylan under the action of the recombinant *Tt*Xyn30A by HPAEC-PAD. The reaction was carried out in 0.05 mM citrate–phosphate buffer pH 4.0 at 50 °C. The substrate and enzyme loadings were 5 mg mL^−1^ and 0.09 U mL^−1^, respectively

The hydrolysis products of beechwood glucuronoxylan under the action of the purified recombinant *Tt*Xyn30A were firstly analyzed by thin layer chromatography (TLC) (Fig. 2). The initial products after 0.1 h of enzymatic reaction seem to be a mixture of UXOS and xylobiose (X2). At longer incubation times the longer chain UXOS were hydrolyzed into shorter chain ones with simultaneous liberation of X2, while xylose was absent. The most prominent hydrolysis products after 24 h of incubation were the MeGlcA^2^Xyl_2_, the MeGlcA^2^Xyl_3_ that were identified by HILIC/ESI-QTOF-MS (superscript states the position of the MeGlcA, while the subscript denotes the number of xylose residues; Additional file 1: Figure S4a, b), and the X2. Moreover, analysis of hydrolysis products by HPAEC-PAD showed no release of other linear XOS except small amounts of X4 that can be attributed to transglycosylation activity (Fig. 3). After incubation with β-xylosidase, only the MeGlcA^2^Xyl_2_ was detected, and the X2 was converted to xylose (Additional file 1: Figure S4c).

**Fig. 4:**
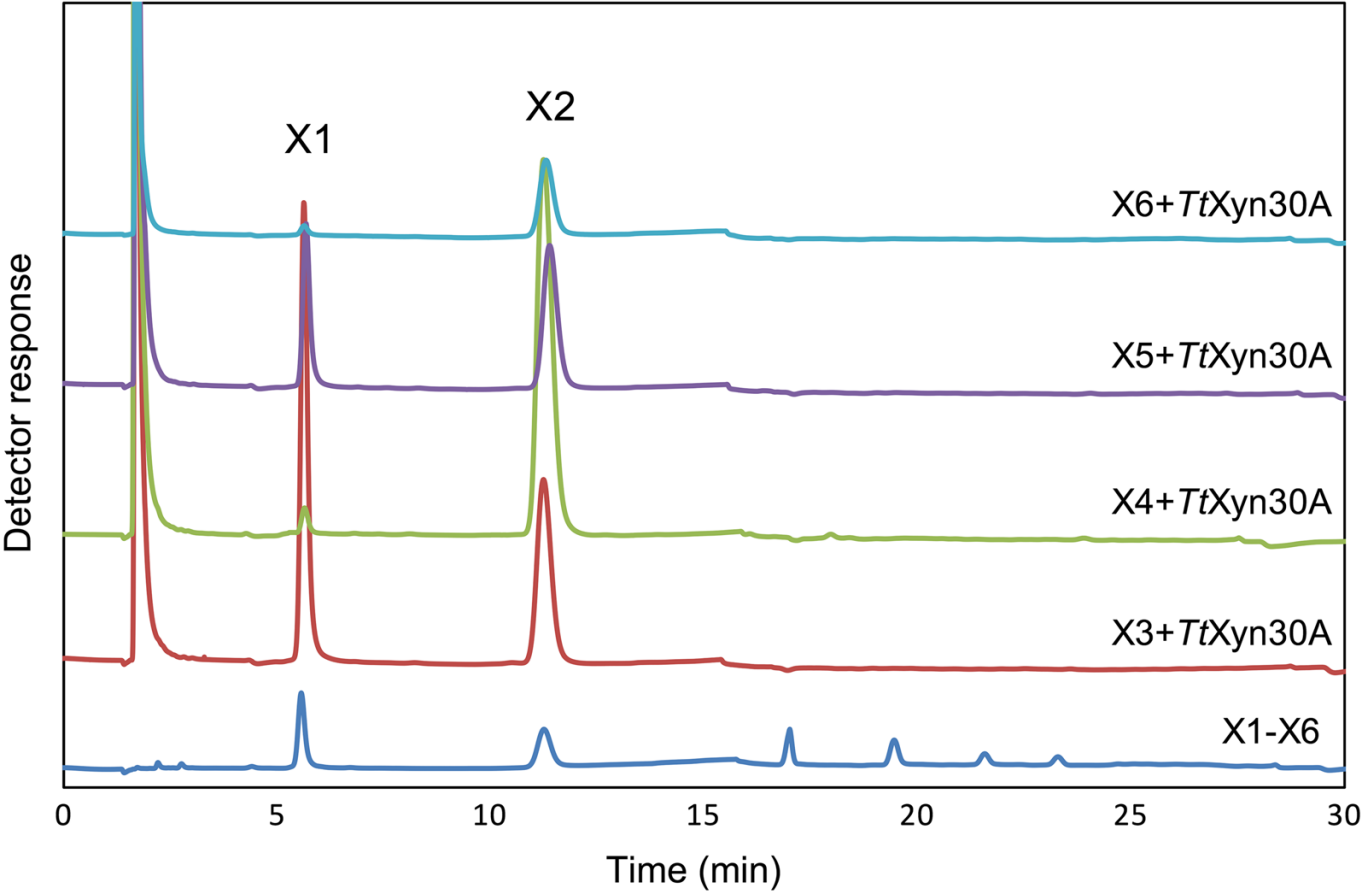
Analysis of the hydrolysis products from xylooligosaccharides (X3–X6) using HPAEC-PAD under the action of *Tt*Xyn30A. The reactions were carried out in 0.05 mM citrate–phosphate buffer pH 4.0 at 50 °C for 18 h, and the enzyme loading was 0.09 U mL^−1^

**Fig. 5:**
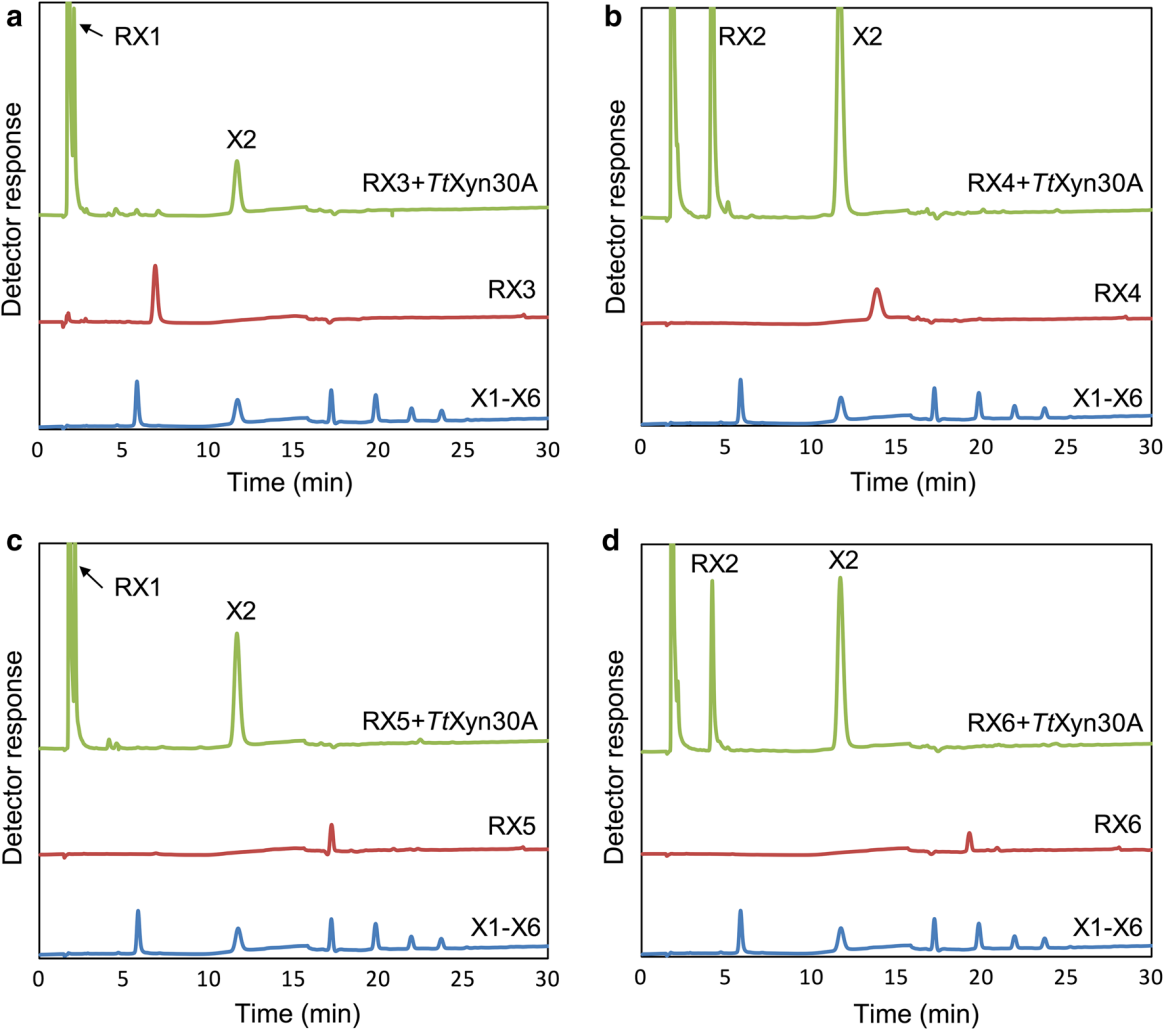
Analysis of the hydrolysis products liberated from NaBH_4_ reduced XOS (RX; DP 3–6) using HPAEC-PAD. Hydrolysis products liberated from **a** RX3, **b** RX4, **c** RX5, and **d** RX6. The reactions were carried out in 0.05 mM citrate–phosphate buffer pH 4.0 at 50 °C and the enzyme loading was 0.09 U mL^−1^

The activity of the recombinant enzyme against linear XOS was also investigated. X3 and X5 were hydrolyzed to xylose and X2, while the hydrolysis of X4 and X6 led to the release of only X2 after 18 h of incubation (Fig. 4). In order to find out whether the enzyme works from the reducing or the non-reducing end, the XOS were treated with NaBH_4_. The NaBH_4_ reduced XOS were incubated with the *Tt*Xyn30A for 18 h revealing the same hydrolysis pattern as in the case of regular XOS (Fig. 5).

Analysis of hydrolysis products by HILIC/ESI-QTOF-MS confirmed the observations that were made by TLC. The main UXOS that were detected up to 1 h of incubation of beechwood glucuronoxylan under the action of *Tt*Xyn30A were MeGlcA^2^Xyl_2_ to MeGlcA^2^Xyl_8_, while X2 was also liberated. After 3 h of enzymatic hydrolysis, MeGlcA^2^Xyl_2_ to MeGlcA^2^Xyl_4_ were detected, while after 24 h of incubation the liberation of only MeGlcA^2^Xyl_2_ and MeGlcA^2^Xyl_3_, alongside X2, was noted (Additional file 2: Figure S1).

## Discussion

The *P. pastoris* expression system was employed to produce the recombinant GH30 xylanase *Tt*Xyn30A from the filamentous ascomycete *T. thermophila*. This specific expression system has been successfully used for the production of two more fungal GH30 xylanases, the XylD from *Bispora* sp. MEY-1 [17] and XynC from *Penicillium purpurogenum* [27]. *P. pastoris* comprise a well-known expression system as its properties such as the efficient enzyme production, the fast growth in inexpensive media, and the appropriate folding and transportation of proteins render it an excellent tool for recombinant protein production. In many other cases, the expression host *E. coli* BL21 has been used for the production of bacterial GH30 xylanases, such as the *St*Xyn30A from *Streptomyces turgidiscabies* [12], the Xyn30A from *Paenibacillus favisporus* [28], the Xyn30D from *Paenibacillus barcinonensis* [11] and the XynA from *Erwinia chrysanthemi* [8] among others. To date, only five fungal GH30 xylanases have been characterized, two from *T. reesei*, one from *Bispora* sp. MEY-1, one from *P. purpurogenum* and recently one from *T. cellulolyticus* [5, 16, 17, 26, 27].

The calculated MW (51.7 kDa) of *Tt*Xyn30A was lower than that determined by SDS-PAGE (61 kDa) possibly due to post-translational modifications. The p*I* determination by IEF revealed multiple protein bands in the range of 5.3–6.2, which was also an indication of protein glycosylation as different carbohydrate groups were added to the molecule. It is common for glycosylation patterns to differ between them and has been reported that proteins expressed in *P. pastoris* exhibited variation concerning the number of the mannose units that were added to the same polysaccharide chain [29]. The treatment of *Tt*Xyn30A with EndoH revealed that the difference in MW and the multiple bands in IEF-PAGE were a result of N-glycosylation. The MW of the treated *Tt*Xyn30A was almost identical with the calculated one, while a single band appeared at pH value 5.6 (Fig. 1a, b).

The hydrolytic activity of the recombinant enzyme was investigated against beechwood glucuronoxylan, wheat arabinoxylan, and other substrates. The purified *Tt*Xyn30A showed the highest activity toward beechwood glucuronoxylan, which consists of a β-1,4-linked xylose backbone decorated with the methylated form of GlcA attached directly to the main chain at xylose C2 (about 13% MeGlcA substitution). However, the specific activity against wheat arabinoxylan, a xylan without MeGlcA substituents along the main chain, was about two orders of magnitude lower than that achieved in the case of beechwood xylan. Bacterial GH30 xylanases are active only against glucuronoxylan, attacking the main chain dependently on the presence of MeGlcA substituents [7, 9]. In contrast, fungal GH30 xylanases seem to exhibit a wider specificity by being active on both glucuronoxylans and arabinoxylans. XynC from *P. purpurogenum* was active on birchwood xylan and rye arabinoxylan exhibiting higher specific activities of 37 U mg^−1^ and 17 U mg^−1^, respectively [27]. Two other fungal GH30 xylanases showed similar specificity. XylD from *Bispora* sp. MEY-1 demonstrated high activity on beechwood xylan and wheat arabinoxylan as well as XYN IV from *T. reesei* [16, 17]. However, the XYN VI from *T. reesei* was the first GH30 xylanase that exhibited catalytic properties that resembled those of bacterial GH30 xylanases [5]. XYN VI showed high specific activity only against xylans containing MeGlcA side substituents, while specific activity less than 0.05 U mg^−1^ was determined for wheat arabinoxylan, and rhodymenan, which is a linear soluble β-1,3-β-1,4-xylan. The catalytic properties that were observed for the *Tt*Xyn30A were similar to those of XYN VI as specific activity of only 0.07 U mg^−1^ was achieved against arabinoxylan after long incubation, unlike beechwood xylan where about 100-fold higher specific activity was noted.

**Fig. 6:**
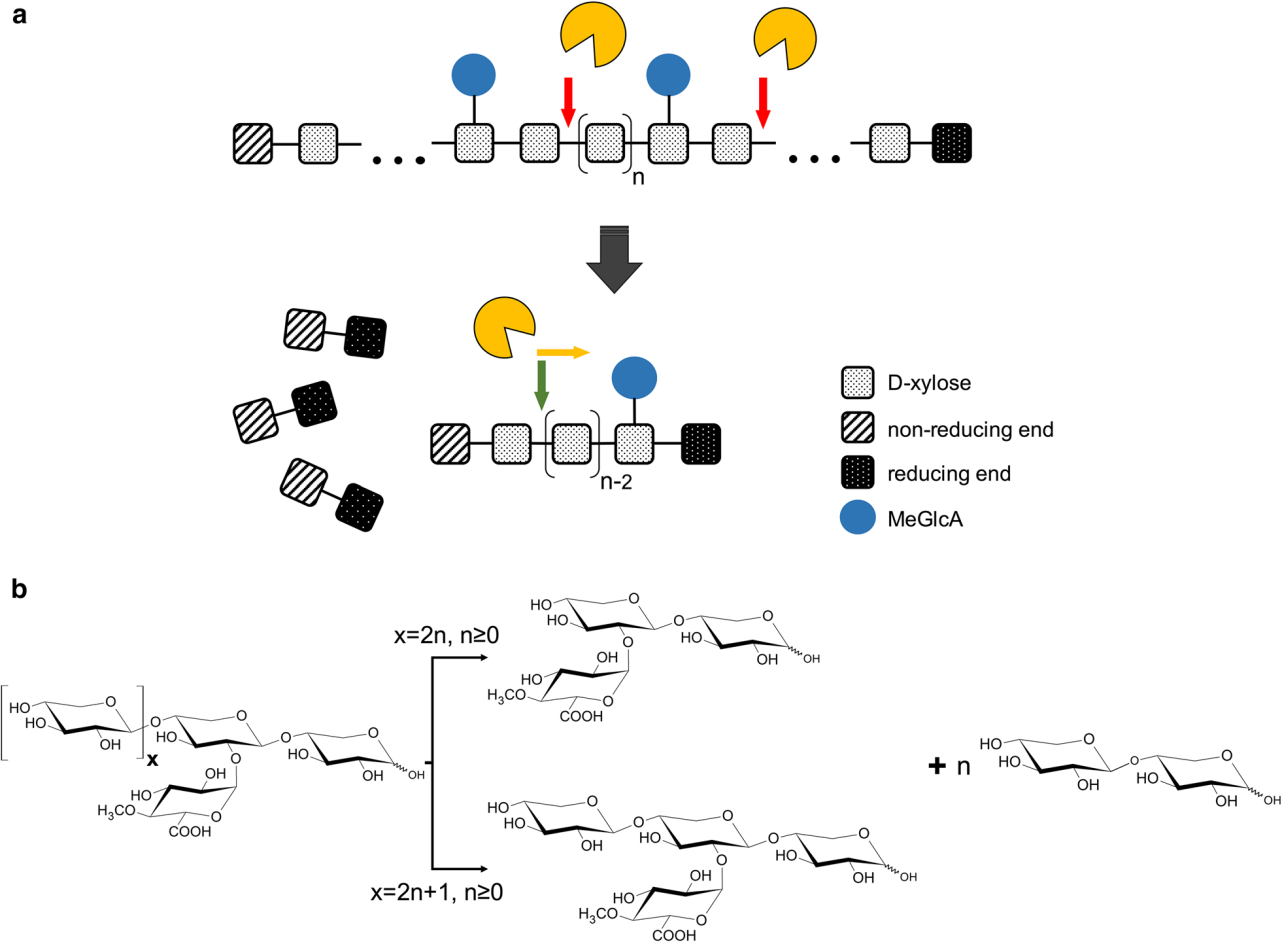
Suggested mode of action of *Tt*Xyn30A against beechwood glucuronoxylan (**a**) and schematic presentation of products liberated from the exo-action of *Tt*Xyn30A against UXOS (**b**). The red arrows indicate the endo-action of the enzyme against the substituted polysaccharide leading to the formation of UXOS. The green arrow indicates the exo-action of the enzyme against the liberated UXOS, while the yellow arrow indicates the direction of the exo-action from the non-reducing end to the reducing end

The incubation of beechwood glucuronoxylan with *Tt*Xyn30A resulted in a mixture of UXOS and X2. Furthermore, the conversion of every generated UXOS to MeGlcA^2^Xyl_2_ upon incubation with a β-xylosidase indicated that the recombinant *Tt*Xyn30A acts on the second glycosidic bond from the MeGlcA substitution and toward the reducing end of the polysaccharide in the same manner as the bacterial GH30s. The major products after longer incubation times were found to be X2, MeGlcA^2^Xyl_2_, and MeGlcA^2^Xyl_3_ (after 24 h of enzymatic hydrolysis). In addition, during the hydrolysis of glucuronoxylan, a slight production of X4 was noted that can be attributed to transglycosylation activity. The formation of such products has been reported before by GH30 xylanases. The production of X4 and X6 has been described during the hydrolysis of glucuronoxylan by the recently characterized Xyn30B [26]. Moreover, the *T. reesei* XYN VI exhibited transglycosylation activity as the hydrolysis of X5 was accompanied by the formation of products of higher degree of polymerization (DP) [5]. The increase in MeGlcA^2^Xyl_2_ concentration after longer incubation indicated the capability of *Tt*Xyn30A to hydrolyze the glycosidic bond to the xylose residue decorated with MeGlcA. The aldouronic acid MeGlcA^2^Xyl_2_ was also one of the major products from glucuronoxylan hydrolysis after long incubation times with XYN VI from *T. reesei* [5]. However, the action of XYN VI against glucuronoxylan also led to the release of a mixture of linear XOS in contrast to the case of *Tt*Xyn30A where only X2 was released. In an attempt to explain the release of only X2 from the generated UXOS during the glucuronoxylan hydrolysis, the activity of *Tt*Xyn30A against linear XOS was investigated. The release of only X2 from X4 and X6, and xylose and X2 from X3 and X5 suggested that the enzyme cleaved X2 units from the XOS chain. The *Tt*Xyn30A exhibited the same hydrolysis pattern against the NaBH_4_ reduced XOS indicating that the enzyme works processively from the non-reducing end of the XOS chain releasing X2. The proposed mode of action is presented in Fig. 6a. The recombinant xylanase initially generates a mixture of UXOS as a result of an endo-action, while due to its xylobiohydrolase activity from the non-reducing end of the generated UXOS, X2 and two specific aldouronic acids are produced. It seems that when xylose residues of UXOS are an even number the main product is MeGlcA^2^Xyl_2_, which was the shortest UXOS from the reaction mixture. In the case when the UXOS contain an odd number of xylose residues the main product is the aldotetrauronic acid MeGlcA^2^Xyl_3_ (Fig. 6b). Similarly, the recently characterized Xyn30B from *T. cellulolyticus* released X2 with simultaneous increase in MeGlcA^2^Xyl_2_ and MeGlcA^2^Xyl_3_ after prolonged incubation with beechwood xylan suggesting an exo-biohydrolase activity [26]. A similar mode of action to these bifunctional xylanases has been observed in processive GH5 endoglucanases, such as Cel5A from *Gloeophyllum trabeum,* revealing the presence of processive cellulases in brown rot fungi [30]. These processive endoglucanases cleave cellulose internally, while releasing soluble oligosaccharides, such as cellobiose, before detaching from polysaccharides. The xylanase XYN IV from *T. reesei* also exhibited both endo- and exo-xylanase activity but in a different manner compared to *Tt*Xyn30A. The XYN IV attacked linear XOS at the first glycosidic linkage from the reducing end liberating xylose, while it did not recognize MeGlcA as substrate specificity determinant. The mode of action of XYN IV suggested that the enzyme aims at liberating xylose from the reducing end of decorated XOS that show resistance to the action of β-xylosidases or other endoxylanases [16].

The optimum activity of *Tt*Xyn30A was observed at 50 °C and pH 4.0. The highest optimum temperature has been reported for *Ct*Xyn30A from *C. thermocellum* (70 °C), while GH30 xylanases from *B. subtilis*, *P. flavisporus*, and *Bispora* sp. possess temperature optima of 65 °C [7, 17, 28, 31]. Unlike bacterial GH30 xylanases (optimal pH at 6.0–6.5), pH optima for the fungal ones are more acidic in the range from pH 3.0 to 5.0.

**Fig. 7:**
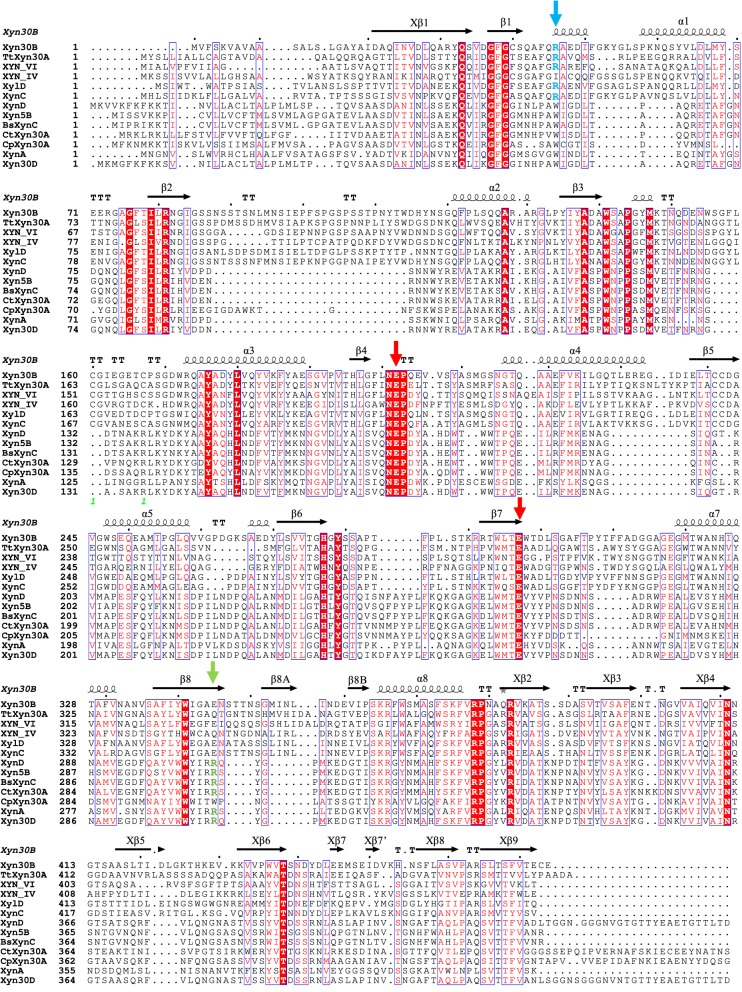
Sequence alignment of *Tt*Xyn30A and other GH30 xylanases, either of bacterial or fungal origin. The R46 residue (as in Xyn30B) is indicated by a *blue arrow*. *Red arrows* indicate the strictly conserved catalytic glutamate residues. The residue R293 of *E. chrysanthemi* XynA is indicated by a *green arrow*. The identical residues are shown in *white on a red background*, while similar residues are shown in *red on a white background*. *Talaromyces cellulolyticus* Xyn30B (GAM36763), *Aeromonas caviae* XynD (AAB63573.1), *Bacillus* sp. BP7 Xyn5B (ADM15019.1), *Bacillus subtilis Bs*XynC (CAA97612.1), *Clostridium thermocellum Ct*Xyn30A (ABN54208.1), *Clostridium papyrosolvens Cp*Xyn30A (EGD48159.1), *Erwinia chrysanthemi* XynA (AAB53151.1), *Paenibacillus barcinonensis* Xyn30D (AEY82463.1), *Bispora* sp. MEY-1 XylD (ADG62369.1), *Penicillium purpurogenum* XynC (AKH40280), *Trichoderma reesei* XYN IV (AAP64786.1), *Trichoderma reesei* XYN VI (G0RV92)

The reveal of 3D crystal structures of GH30 xylanases is of utmost importance in order to elucidate the interaction between these enzymes and their substrate; especially in the case of bacterial GH30 xylanases where the recognition of the GlcA or MeGlcA substituent is crucial for their activity. To this day, 3D structures for five bacterial GH30 xylanases, members of subfamily 8, have been determined and more specifically for XynC from *B. subtilis* [32], XynA from *E. chrysanthemi* [33], *Cp*Xyn30A from *C. papyrosolvens* [14], Xyn30D from *P. barcinonensis* [34], and *Ct*Xyn30A from *C. thermocellum* [35]. Structural studies of XynA from *E. chrysanthemi*, crystallized with the aldotetrauronic acid as a ligand, revealed that a conserved arginine residue (R293) interacts with the carboxyl group of MeGlcA (ionic interaction) side substituent and contributes this way to the substrate recognition [33]. Additionally, in the case of *St*Xyn30A from *S. turgidiscabies*, the catalytic residue R296 that is conserved at subsite − 2 was replaced and as a result, the activity of the mutant constructs against glucuronoxylan was essentially reduced [12]. The only characterized bacterial GH30 xylanase that does not conserve this specific amino acid residue is *Cp*Xyn30A from *C. papyrosolvens* by having a tryptophan residue [14]. However, none of the six characterized to date fungal GH30 xylanases seem to conserve this arginine residue (green arrow; Fig. 7). During the publication process of the present study, the first crystal structure of a member of GH30_7 subfamily was reported, giving an insight on the MeGlcA recognition of the fungal xylanase counterparts. The 3D structure of Xyn30B from *T*. *cellulolyticus*, alongside site directed mutagenesis, revealed that the R46 residue plays a key role for the enzyme’s apendage-dependent endo-mode of action against xylan [26]. The R46 residue, which is R51 in *Tt*Xyn30B, is conserved in the amino acid sequences of all characterized GH30_7 glucuronoxylanases. However, XYN IV, which does not require the MeGlcA as substrate specificity determinant, does not have the arginine residue (I52 in XYN IV; Fig. 7). The overall structure of Xyn30B is similar to the bacterial xylanases, members of GH30_8 subfamily, which is composed of a (α/β)_8_-catalytic domain formed by 8 α-helices and β strands together with a small β-rich domain consisting of 9 β-strands [26].

The catalytic glutamate residues (E165 and E253 in XynA) are conserved in all GH30 sequences. The glutamate that acts as a nucleophile in *Tt*Xyn30A sequence is located in the position 205, while the catalytic acid/base residue is E295. Both E205 and E295 are located in conserved regions. The amino acids responsible for hydrogen bonding between XynA and MeGlcA were Y255, S258, W289, and Y295 of which only the W289 is conserved in *Tt*Xyn30A in position 338 or in position 341 for the Xyn30B. The ligand’s xylose unit (− 1) in XynA 3D structure interacts with W113 and N164 in subsite − 1 through hydrogen bonds. Both of these amino acid residues are conserved in all GH30 xylanases sequences, either of bacterial or fungal origin. The aromatic ring of W289 is also responsible for stacking interactions with the xylose unit of ligand in subsite − 1 (W338 in *Tt*Xyn30A). The amino acids that form the subsite − 2b of Xyn30B are conserved in *Tt*Xyn30A (R51, L299, W338, I339, T346, and S348) except for Xyn30B E345, which is Q342 (Fig. 7).

## Conclusions

In summary, the present study reports the heterologous expression and characterization of a fungal GH30 xylanase designated *Tt*Xyn30A from *T. thermophila.* The catalytic properties of *Tt*Xyn30A were investigated and revealed an endo-enzymatic activity that was dependent on the MeGlcA side substituent. However, the recombinant xylanase also exhibited an exo-activity against linear and decorated XOS releasing X2 units from the non-reducing end. The presented mode of action is in accordance with the recently discovered Xyn30B from *T. cellulolyticus*, rendering the *Tt*Xyn30A a novel GH30 xylanase with xylobiohydrolase auxiliary activity. Therefore, the difference between the catalytic properties of fungal and bacterial GH30 xylanases or even among fungal ones is not trivial. Crystal studies of *Tt*Xyn30A with an aldouronic acid as ligand are underway, as well as synergistic experiments with other hemicellulases, aiming in the understanding of the role of these unique xylanases in plant cell wall degradation.

## Methods

### Enzymes and substrates

EasySelect™ *Pichia* Expression kit and pPICZα plasmid vectors were acquired from Invitrogen (Waltham, MA, USA). The restriction enzymes *Cla*I, *Xba*I, and *Sac*I were purchased from Takara Bio Inc. (Kusatsu, Shiga Prefecture, Japan). Gel extraction and plasmid mini prep kits were both obtained from Macherey–Nagel (Düren, Germany). Beechwood xylan, wheat arabinoxylan, carob galactomannan, konjac glucomannan, barley β-glucan, and xylooligosaccharides (XOS) (DP 2–6) were purchased from Megazyme (Bray, Co. Wicklow, Ireland). Aldotetrauronic (terminal substitution, borohydride reduced) and aldopentauronic acids (internal substitution, borohydride reduced), and β-xylosidase from *Bacillus pumilus* were a kind gift from Megazyme. Endoglycosidase H was from New England Biolabs (Ipswich, MA, USA).

### Enzyme cloning and production

The gene coding for the putative protein *Tt*Xyn30A (Protein ID 38558; chromosome 1: 7956351–7958140; accession no. XP_003660270.1), designated *ex30a*, was synthesized as codon optimized for expression in *P. pastoris* host strain X-33 and was cloned in vector pEX-K4 by GenScript (Piscataway, NJ, USA). The pPICZαC *Pichia* vector accomplished the secreted expression of *Tt*Xyn30A. The pEX-K4-*ex30a* vector was digested with the restriction enzymes *Cla*I and *Xba*I and then the gel-purified fragment was inserted at the corresponding sites into the plasmid vector pPICZαC in frame with both the yeast α-secretion factor and the C-term-His_6_ tag encoding sequences. The resulting recombinant pPICZαC/*ex30a* construct was multiplied using the chemically competent *Escherichia coli* One Shot^®^ Top10 (Invitrogen) cells and subsequently, the successfully transformed clones were selected based on Zeocin™ resistance (25 μg mL^−1^). Before the transformation of the *ex30a* gene into *P. pastoris* by electroporation, restriction analysis, and DNA sequencing assured its presence in the recombinant vector. *P. pastoris* was grown in shake flasks at 30 °C in a rich buffered glycerol-complex medium (BMGY) or methanol (0.5%, v/v) containing buffered methanol-complex medium (BMMY) (for induction), as detailed by EasySelect™ *Pichia* Expression kit. For maintaining *P. pastoris* cultures and plates, yeast extract-peptone-dextrose (YPD) medium was used, while for transformants selection, YPD plates were supplemented with sorbitol (YPDS) and Zeocin™ at a final concentration of 1 M and 100 μg mL^−1^, respectively.

#### Enzyme purification

Immobilized metal-ion affinity chromatography (IMAC) was used in order to purify the recombinant enzyme *Tt*Xyn30A after its production in *P. pastoris* cultures as described previously [36]. Culture supernatants were concentrated by ultrafiltration and the concentrates, after they were equilibrated with 0.02 M Tris–HCl buffer (pH 8.0) containing 0.3 M NaCl, were purified using an IMAC column (1 × 15 cm, Talon; Clontech, Mountain View, CA, USA). Sodium dodecyl sulfate polyacrylamide gel electrophoresis (SDS-PAGE) and isoelectric focusing (IEF) were employed in order to determine the molecular weight (MW) and the isoelectric point (p*I*) of the purified recombinant protein, respectively, as detailed previously [37]. The purified enzyme was examined for N-glycosylation through Endoglycosidase H treatment under denaturing conditions according to the manufacturer’s instructions. Deglycosylated samples were analyzed by both SDS-PAGE and IEF. The protein concentration of the purified enzyme was determined by measuring *A*_280_ using the recombinant xylanase’s molar extinction absorptivity as it was estimated by ProtParam tool [38, 39].

#### Enzymatic activity assays

The enzyme activity was assayed against 0.5% (w/v) beechwood xylan, for 30 min in 0.05 M citrate–phosphate buffer pH 5.0. Reducing sugars that were released by beechwood xylan hydrolysis, were determined by the 3,5-dinitrosalicylic acid (DNS) method using xylose for constructing the calibration curve [40]. One unit (U) of xylanase activity was defined as the amount of enzyme that released 1 μmol of reducing sugars per minute under standard assay conditions.

#### Biochemical characterization

The optimal temperature was determined under the standard assay procedure at temperatures ranging from 20 to 80 °C in 0.05 M citrate–phosphate buffer (pH 5.0), while the optimal pH was determined at the optimal temperature over the pH range from 3.0 to 7.0. The pH stability was determined after incubating the enzyme in 0.1 M citrate–phosphate (pH 3.0–7.0), 0.1 M Tris–HCl (pH 8.0–9.0), and 0.1 M glycine–NaOH (pH 10.0–11.0) at 4 °C for 24 h and then measuring the residual activity employing the standard enzymatic activity assay.

The effect of various metal ions or chemical compounds (Ca^2+^, Co^2+^, Ni^2+^, Zn^2+^, Na^+^, K^+^, Cu^2+^, Mg^2+^, Mn^2+^, Fe, EDTA, Urea, SDS) on the enzyme activity was determined by incubating the enzyme in the presence of 1, 5, and 10 mM of the individual compounds in 0.1 M Tris–HCl buffer pH 8.0 at room temperature for 2 h. The residual activity was then measured under standard assay conditions, in the presence of metal ions or chemical reagents, and compared to the activity of the untreated enzyme, which was taken as control (100%).

To investigate the substrate specificity of *Tt*Xyn30A, multiple substrates, such as beechwood xylan, wheat arabinoxylan, carob galactomannan, konjac glucomannan, and barley β-glucan were selected. Enzyme activity was determined after incubation in 0.05 M citrate–phosphate buffer (pH 4.0) containing 0.5% (w/v) of each substrate at 50 °C for 30 min. The initial velocity of the recombinant *Tt*Xyn30A was measured at 50 °C in 0.05 M citrate–phosphate buffer (pH 4.0) with beechwood xylan concentrations varying from 1 to 10 mg mL^−1^. Data were fitted to the Michaelis–Menten equation to estimate the values *K*_m_, *V*_max_, and *K*_cat_, using GraphPad Prism version 6, GraphPad Software (La Jolla, CA, USA).

### Analysis of enzymatic hydrolysis products

Hydrolysis products of beechwood xylan under the action of recombinant *Tt*Xyn30A were analyzed by TLC. Reaction conditions were pH 4.0 at 50 °C, substrate concentration was 0.5% (w/v) and enzyme loading was 0.09 mg mL^−1^. Samples were taken at certain time intervals (0.1, 0.5, 1.0, 3.0, and 24.0 h) of the enzymatic reaction and were analyzed by TLC using silica gel 60 F_254_ plates (Merck, Darmstadt, Germany) developed twice in a solvent mixture of ethyl acetate/2-propanol/acetic acid/formic acid/water (25:10:5:1:15 v/v). Visualization of the developed sugars was performed by spraying the TLC plates with 6.5 mM *N*-(1-naphthyl)ethylenediamine dihydrochloride reagent in methanol containing 3% sulfuric acid [41] followed by heating at 100 °C for 10 min.

The analysis of the hydrolysis products was also performed employing hydrophilic interaction liquid chromatography/electrospray ionization-quadrupole time-of-flight mass spectrometry (HILIC/ESI-QTOF-MS) as described previously [42]. The separation was performed at 40 °C on an ACQUITY UPLC BEH Amide column (2.1 × 100 mm, 1.7 μm; Waters Dublin, Ireland) equipped with a guard column of the same packaging material. In the case of positive ionization mode (PI) the aqueous phase consisted of H_2_O with 1 mM ammonium formate and 0.01% formic acid and the organic phase was CH_3_CN/H_2_0 95/5 with 1 mM ammonium formate and 0.01% formic acid, while for negative ionization mode (NI) the aqueous phase consisted of H_2_O with 10 mM ammonium formate and the organic phase was CH_3_CN/H_2_0 95/5 with 10 mM ammonium formate. The elution gradient, for both ionization modes, started with 100% of organic phase for 2 min, decreasing to 5% in 10 min, and kept constant for 5 min. The initial conditions were restored within 0.1 min and let to re-equilibrate for 8 min. The flow rate was 0.2 mL min^−1^ and the injection volume was 5 μL. The QTOF system was equipped with an ESI, operating in PI and NI mode employing the following operation parameters: capillary voltage 2500 V (PI) and 3500 (NI); end plate offset, 500 V; nebulizer pressure 2 bar; drying gas 8 L min^−1^, and gas temperature 200 °C.

Hydrolysis of XOS (DP 3–6) was conducted in 0.05 M citrate–phosphate pH 4.0 using an enzyme load of 0.09 mg mL^−1^ at 50 °C for up to 18 h. To evaluate the exo-action of *Tt*Xyn30A from the non-reducing end, the XOS were treated with sodium borohydride (NaBH_4_) and the NaBH_4_ reduced XOS were incubated with the enzyme as described for the hydrolysis of untreated XOS. To determine the hydrolysis products, the enzymatic reactions were boiled to inactivate the enzyme and then subjected to high performance anion exchange chromatography (HPAEC) equipped with an ED40 pulsed amperometric detector (PAD) (Dionex, Sunnyvale, CA, USA) using a CarboPac PA-1 (4 × 250 mm) column. The elution conditions were as follows: 0–5 min, 100 mM NaOH (100%); 5.1–30 min, NaOAc (500 mM) in 100 mM NaOH (0–20%); 30.1–35 min, NaOAc (500 mM) in 100 mM NaOH (100%); 35.1–50 min, 100 mM NaOH (100%).

### Protein sequence analysis

The signal peptide sequence was predicted using the server SignalP 4.0 [43]. N-glycosylation and O-glycosylation sites were analyzed and predicted by NetNGlyc 1.0 and NetOGlyc 4.0 servers [44, 45]. Multiple sequence alignment of GH30 xylanases was performed using the Clustal Omega program [46] and the visualization was conducted employing the ESPript server [47].

## Additional files

**Additional file 1: Figure S1.** Effect of pH (a) and temperature (b) on the activity of *Tt*Xyn30A against beechwood xylan. Each data point represents the mean ± SD (*n *= 3). **Figure S2.** Effect of pH on the stability of *Tt*Xyn30A. Each data point represents the mean ± SD (*n *= 2). **Figure S3.** Rate of hydrolysis of beechwood xylan by *Tt*Xyn30A. Total reducing sugars were expressed as xylose equivalents. The substrate and enzyme loadings were 5 mg mL^−1^ and 0.09 U mL^−1^, respectively. Each data point represents the mean ± SD (*n *= 2). **Figure S4.** Determination of hydrolysis products (a) MeGlcA^2^Xyl_2_, and (b) MeGlcA^2^Xyl_3_ from beechwood glucuronoxylan under the action of *Tt*Xyn30A after 24 h of incubation using HILIC-ESI-QTOFMS. (c) Determination of MeGlcA^2^Xyl_2_ after incubation of generated UXOS with β-xylosidase. **Table S1.** Effect of metal ions or chemical compounds on relative activity of the recombinant *Tt*Xyn30A.

**Additional file 2: Figure S1.** HILIC/ESI-QTOF-MS analysis of hydrolysis products liberated from beechwood glucuronoxylan after a (a) 10 min, (b) 30 min, (c) 1 h, (d) 3 h, and (e) 24 h incubation with the *Tt*Xyn30A. The reaction was carried out in 0.05 mM citrate–phosphate buffer pH 4.0 at 50 °C. The substrate and enzyme loadings were 5 mg mL^−1^ and 0.09 U mL^−1^, respectively.

## Data Availability

All data generated or analyzed during this study are included in this published article and its additional information files.

## Abbreviations

BMGY: buffered glycerol-complex media
BMMY: buffered methanol-complex media
DNS: 3,5-dinitrosalicylic acid
DP: degree of polymerization
GH30: glycoside hydrolase family 30
GlcA: glucuronic acid
HILIC/ESI-QTOF-MS: liquid chromatography/electrospray ionization-quadrupole time-of-flight mass spectrometry
HPAEC-PAD: high performance anion exchange chromatography-pulsed amperometric detector
IEF-PAGE: isoelectric focusing polyacrylamide gel electrophoresis
IMAC: immobilized metal-ion affinity chromatography
MeGlcA: 4-*O*-methyl-d-glucuronic acid
MeGlcA^2^Xyl_2_: aldotriuronic acid
MeGlcA^2^Xyl_3_: aldotetrauronic acid
MW: molecular weight
SDS-PAGE: sodium dodecyl sulfate polyacrylamide gel electrophoresis
TLC: thin layer chromatography
UXOS: uronic xylooligosaccharides
XOS: xylooligosaccharides
YPD: yeast extract-peptone-dextrose media
YPDS: yeast extract-peptone-dextrose-sorbitol media
