# Supplementary material for: A novel fungal GH30 xylanase with xylobiohydrolase auxiliary activity
Source: Biotechnol Biofuels. 2019 May 11;12:120. doi: 10.1186/s13068-019-1455-2 (PMC6511221; doi:10.1186/s13068-019-1455-2)
Supplement: Supplementary file 1 — Additional file 1: Figure S1. Effect of pH (a) and temperature (b) on the activity of TtXyn30A against beechwood xylan. Each data point represents the mean ± SD (n = 3). Figure S2. Effect of pH on the stability of TtXyn30A. Each data point represents the mean ± SD (n = 2). Figure S3. Rate of hydrolysis of beechwood xylan by TtXyn30A. Total reducing sugars were expressed as xylose equivalents. The substrate and enzyme loadings were 5 mg mL−1 and 0.09 U mL−1, respectively. Each data point represents the mean ± SD (n = 2). Figure S4. Determination of hydrolysis products (a) MeGlcA2Xyl2, and (b) MeGlcA2Xyl3 from beechwood glucuronoxylan under the action of TtXyn30A after 24 h of incubation using HILIC-ESI-QTOFMS. (c) Determination of MeGlcA2Xyl2 after incubation of generated UXOS with β-xylosidase. Table S1. Effect of metal ions or chemical compounds on relative activity of the recombinant TtXyn30A. [file 13068_2019_1455_MOESM1_ESM.docx]

**Additional file 1**

**Title:** A novel appendage-dependent fungal GH30 xylanase with xylobiohydrolase side activity

**Authors:** Constantinos Katsimpouras^1^, Grigorios Dedes^1^, Nikolaos S. Thomaidis^2^, Evangelos Topakas^1,3*^

**Affiliations:**

^1^*Industrial Biotechnology & Biocatalysis Group, School of Chemical Engineering, National Technical University of Athens, 9 Iroon Polytechniou Str., Zografou Campus, Athens 15780, Greece.*

^2^*Laboratory of Analytical Chemistry, Department of Chemistry, National and Kapodistrian University of Athens, Panepistimioupolis Zografou, 15771, Athens, Greece*

*^3^Biochemical and Chemical Process Engineering, Division of Sustainable Process Engineering, Department of Civil, Environmental and Natural Resources Engineering, Luleå University of Technology, SE-97187 Luleå, Sweden.*

**Corresponding author**; E-mail: [vtopakas@chemeng.ntua.gr](mailto:vtopakas@chemeng.ntua.gr)


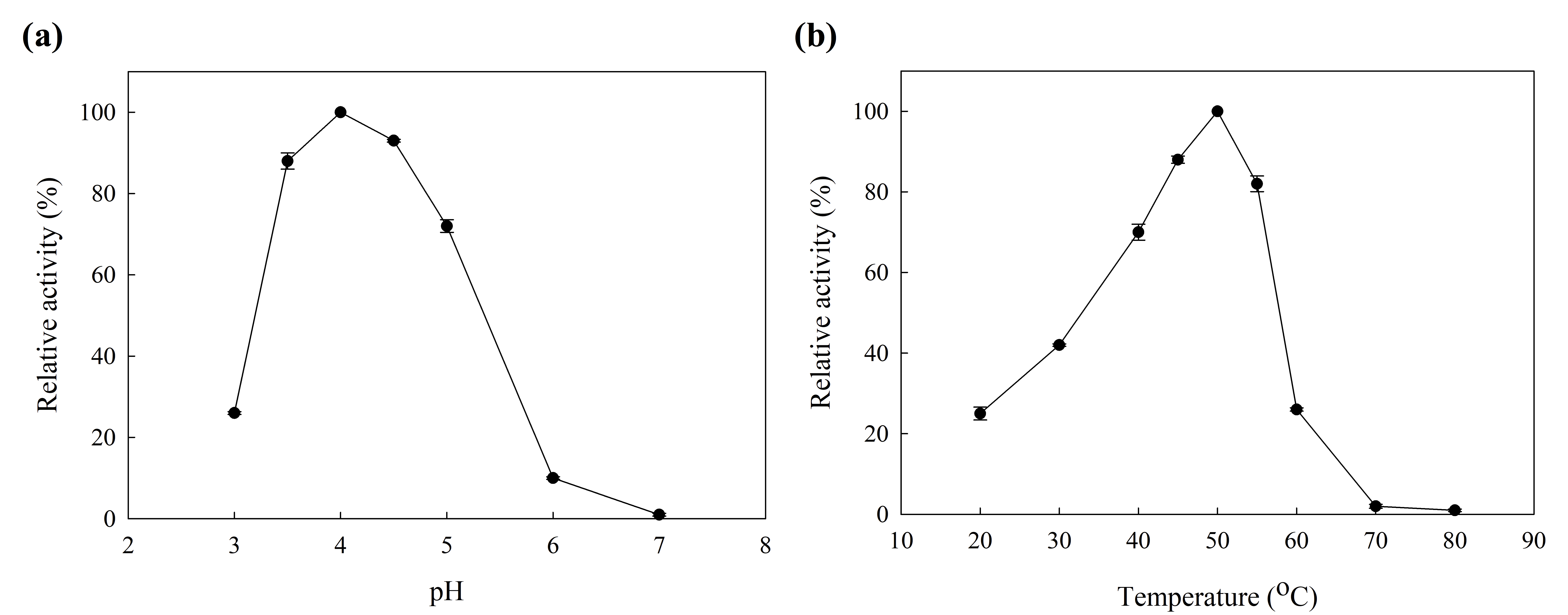


**Figure S1:** Effect of pH (a) and temperature (b) on the activity of *Tt*Xyn30A against beechwood xylan. Each data point represents the mean ± SD (*n*=3).


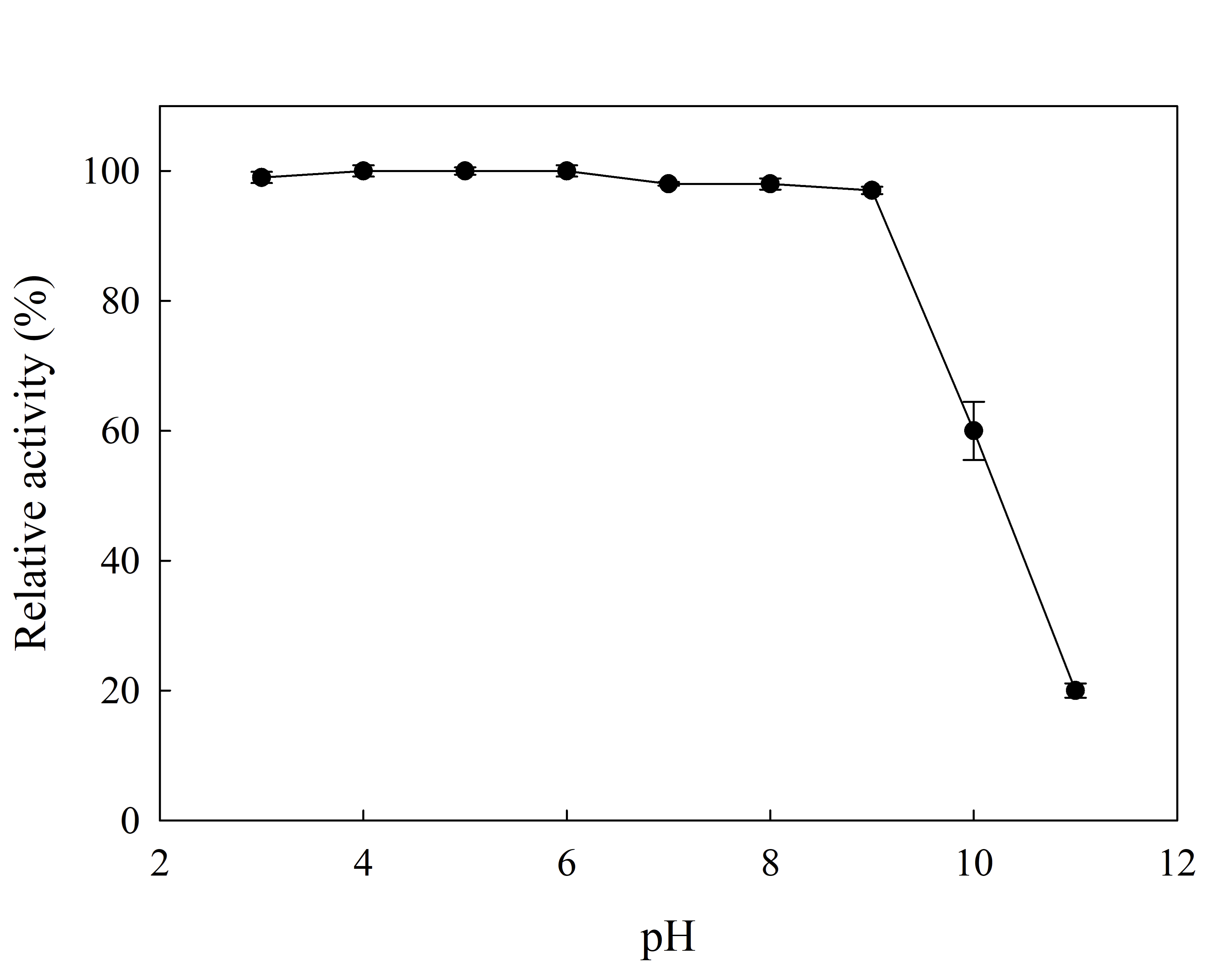


**Figure S2:** Effect of pH on the stability of *Tt*Xyn30A. Each data point represents the mean ± SD (*n*=2).


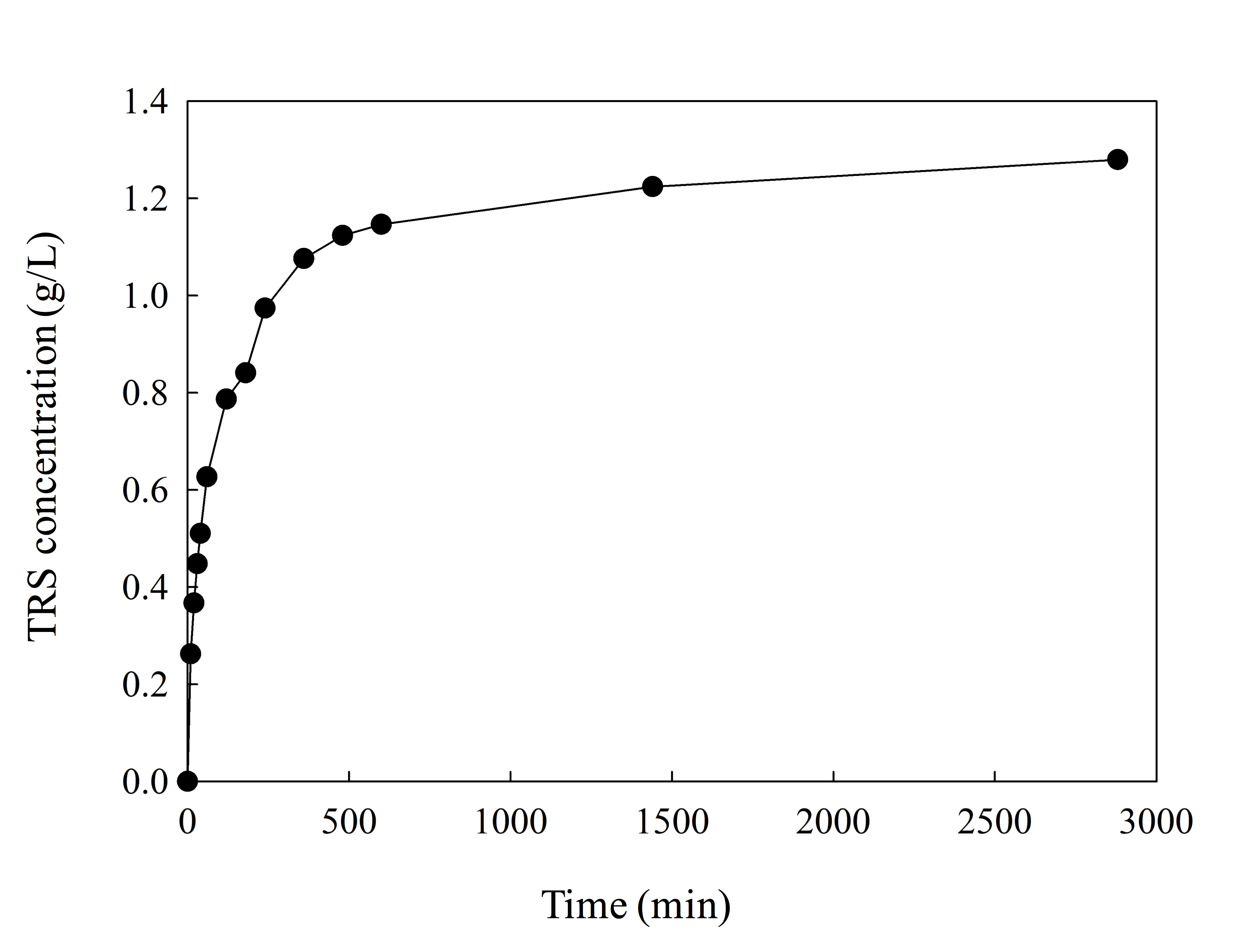


**Figure S3:** Rate of hydrolysis of beechwood xylan by *Tt*Xyn30A. Total reducing sugars were expressed as xylose equivalents. The substrate and enzyme loadings were 5 mg·mL^-1^ and 0.09 U·mL^-1^, respectively. Each data point represents the mean ± SD (*n*=2).

**
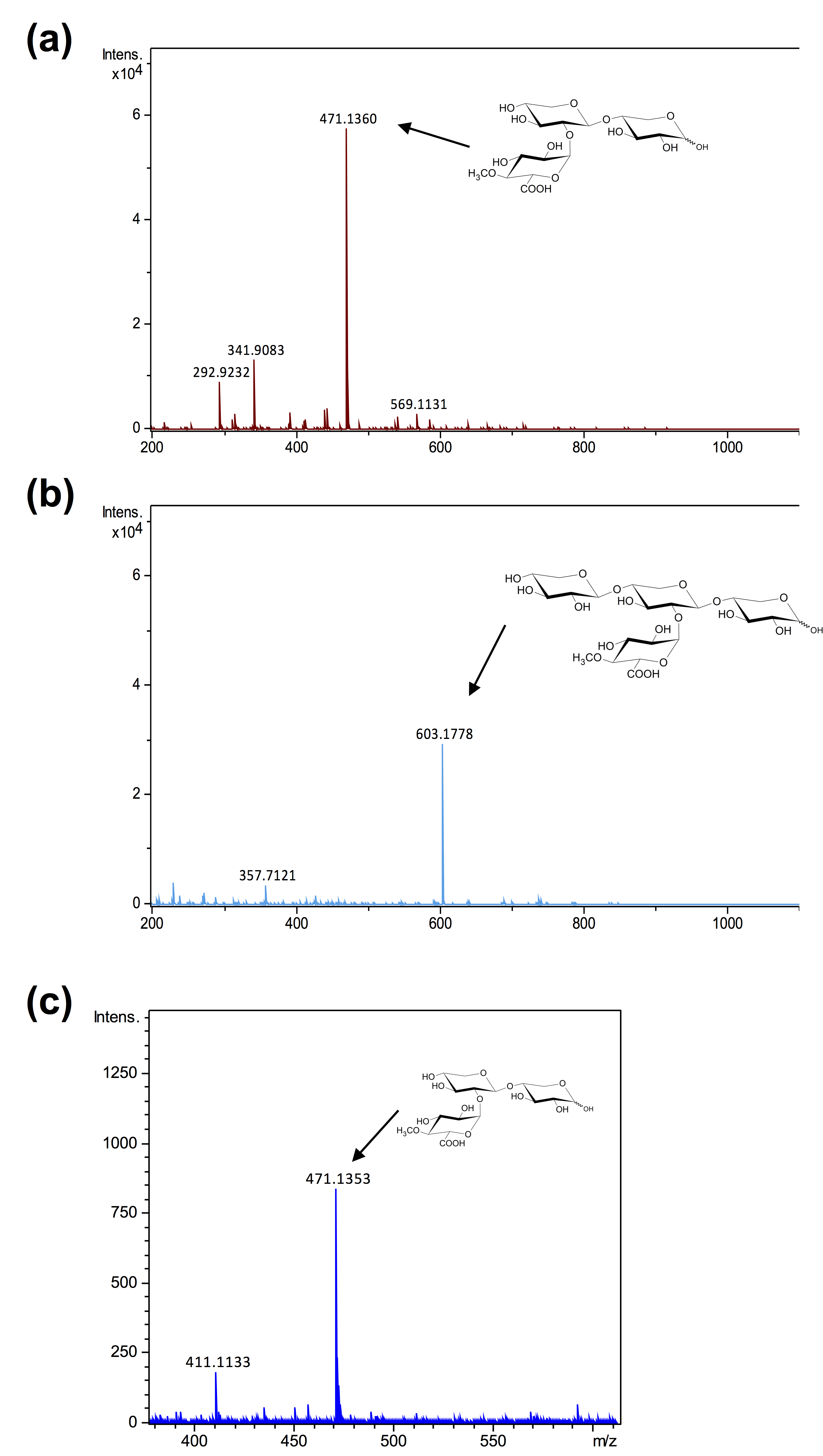
**

**Figure S4:** Determination of hydrolysis products (a) MeGlcA^2^Xyl_2_, and (b) MeGlcA^2^Xyl_3_ from beechwood glucuronoxylan under the action of *Tt*Xyn30A after 24 h of incubation using HILIC-ESI-QTOFMS. (c) Determination of MeGlcA^2^Xyl_2_ after incubation of generated UXOS with β-xylosidase.

**Table S1.** Effect of metal ions or chemical compounds on relative activity of the recombinant *Tt*Xyn30A.

| **Metal ions or chemical compounds** | **Relative activity (%)** | | |
| --- | --- | --- | --- |
|  | **1 mM** | **5 mM** | **10 mM** |
| - | 100.0 | 100.0 | 100.0 |
| Na^+^ | 104.3±4.2 | 102.0±0.4 | 98.3±4.2 |
| K^+^ | 108.6±1.7 | 104.9±1.6 | 98.3±3.0 |
| Ca^2+^ | 110.0±1.3 | 105.3±0.2 | 98.3±3.0 |
| Co^2+^ | 117.0±1.7 | 123.8±0.0 | 141.5±4.9 |
| Ni^2+^ | 108.1±1.5 | 102.7±0.6 | 101.9±3.3 |
| Cu^2+^ | 106.5±0.7 | 112.8±1.5 | 112.8±0.1 |
| Mg^2+^ | 107.0±0.4 | 99.1±1.6 | 89.8±2.5 |
| Fe^3+^ | 109.1±2.5 | 103.3±2.7 | 94.9±1.2 |
| Mn^2+^ | 135.3±3.7 | 136.6±1.5 | 143.8±1.5 |
| Zn^2+^ | 107.0±4.5 | 96.0±1.6 | 90.9±2.7 |
| SDS | 50.2±1.3 | 21.8±1.2 | 2.1±0.6 |
| EDTA | 103.4±5.4 | 93.7±0.4 | 85.3±2.2 |
| Urea | 103.9±3.6 | 98.1±0.2 | 90.9±0.4 |
