# Supplementary material for: A novel fungal GH30 xylanase with xylobiohydrolase auxiliary activity
Source: Biotechnol Biofuels. 2019 May 11;12:120. doi: 10.1186/s13068-019-1455-2 (PMC6511221; doi:10.1186/s13068-019-1455-2)
Supplement: Supplementary file 2 — Additional file 2: Figure S1. HILIC/ESI-QTOF-MS analysis of hydrolysis products liberated from beechwood glucuronoxylan after a (a) 10 min, (b) 30 min, (c) 1 h, (d) 3 h, and (e) 24 h incubation with the TtXyn30A. The reaction was carried out in 0.05 mM citrate–phosphate buffer pH 4.0 at 50 °C. The substrate and enzyme loadings were 5 mg mL−1 and 0.09 U mL−1, respectively. [file 13068_2019_1455_MOESM2_ESM.docx]

**Additional file 2**

**Title:** A novel appendage-dependent fungal GH30 xylanase with xylobiohydrolase side activity

**Authors:** Constantinos Katsimpouras^1^, Grigorios Dedes^1^, Nikolaos S. Thomaidis^2^, Evangelos Topakas^1,3*^

**Affiliations:**

^1^*Industrial Biotechnology & Biocatalysis Group, School of Chemical Engineering, National Technical University of Athens, 9 Iroon Polytechniou Str., Zografou Campus, Athens 15780, Greece.*

^2^*Laboratory of Analytical Chemistry, Department of Chemistry, National and Kapodistrian University of Athens, Panepistimioupolis Zografou, 15771, Athens, Greece*

*^3^Biochemical and Chemical Process Engineering, Division of Sustainable Process Engineering, Department of Civil, Environmental and Natural Resources Engineering, Luleå University of Technology, SE-97187 Luleå, Sweden.*

**Corresponding author**; E-mail: [vtopakas@chemeng.ntua.gr](mailto:vtopakas@chemeng.ntua.gr)

**(a)**

**(b)**

**(c)**

**(d)**

**(e)**

**Figure S1:** HILIC/ESI-QTOF-MS analysis of hydrolysis products liberated from beechwood glucuronoxylan after a **(a)** 10 min, **(b)** 30 min, **(c)** 1 h, **(d)** 3 h, and **(e)** 24 h incubation with the *Tt*Xyn30A. The reaction was carried out in 0.05 mM citrate-phosphate buffer pH 4.0 at 50 °C. The substrate and enzyme loading were 5 mg·mL-1 and 0.09 U·mL-1, respectively.
